# Molecular characterisation of virulence graded field isolates of myxoma virus

**DOI:** 10.1186/1743-422X-7-49

**Published:** 2010-02-26

**Authors:** Kevin P Dalton, Ines Nicieza, Aroa Baragaño, Jose Manuel Martín Alonso, Francisco Parra

**Affiliations:** 1 Instituto Universitario de Biotecnología de Asturias, Departamento de Bioquímica y Biología Molecular, Edificio Santiago Gascón, Campus El Cristo, Universidad de Oviedo, 33006 Oviedo, España

## Abstract

**Background:**

*Myxoma virus *(MV) has been endemic in Europe since shortly after its deliberate release in France in 1952. While the emergence of more resistant hosts and more transmissible and attenuated virus is well documented, there have been relatively few studies focused on the sequence changes incurred by the virus as it has adapted to its new host. In order to identify regions of variability within the MV genome to be used for phylogenetic studies and to try to investigate causes of MV strain attenuation we have molecularly characterised nine strains of MV isolated in Spain between the years 1992 and 1995 from wide ranging geographic locations and which had been previously graded for virulence by experimental infection of rabbits.

**Results:**

The findings reported here show the analysis of 16 genomic regions accounting for approximately 10% of the viral genomes. Of the 20 genes analysed 5 (M034L, M069L, M071L, M130R and M135R) were identical in all strains and 1 (M122R) contained only a single point mutation in an individual strain. Four genes (M002L/R, M009L, M036L and M017L) showed insertions or deletions that led to disruption of the ORFs.

**Conclusions:**

The findings presented here provide valuable tools for strain differentiation and phylogenetic studies of MV isolates and some clues as to the reasons for virus attenuation in the field.

## Background

*Myxoma virus *(MV) causes myxomatosis in the European rabbit (*Oryctolagus cuniculus*). The disease is a recurrent problem in rabbit farms and in wild rabbit populations throughout Europe [1-4]. Due to the unique manner of its introduction into the European rabbit population, MV provides an excellent model for studying the coevolution of a virus and its host. The complex virus/host relationship has been shown to lead to the emergence of more transmissible/attenuated virus strains and more resistant hosts (for review see [5]). Such studies have been carried out using experimental rabbit infections, however, few studies have characterised the sequence changes incurred by the virus during adaptation to its new host [1,6-10].

MV strains are classified into 5 virulence grades (I to V, I being most virulent and V the most attenuated) based on the mean survival time of rabbits after infection [10]. Due to the large size of the viral genome (161.8 kb) restriction fragment length polymorphism (RFLP) analysis of purified viral genomes has been the traditional method for the molecular differentiation of MV strains [6,11]. However, no correlation between RFLPs and attenuation has been demonstrated [6].

Recent studies of field strains of MV have focussed on sequencing genes or gene fragments, however, the majority of these strains have not been characterised for virulence in rabbits [1,9]. Epidemiological studies on large numbers of virus isolates are essential to differentiate the types of circulating MV-isolates and to identify new emergent strains which might have different degrees of virulence.

Hampered by the large size of the viral genome, to date only two full-length genome sequences are available, strain Lausanne (Lu) [12] and strain 6918 [8]. These data allowed the first direct comparison between two full-length MV genomes, one (Lu) being wild-type (wt) and the other (6918) being attenuated [8].

Strain 6918 was first isolated during field sampling of MV in Spain [13]. In that study twenty isolates, collected from wide ranging locations between 1992 and 1995, were further characterized for their virulence grade and horizontal transmission in experimental infections of rabbits. These MV-isolates included a representative example of all five virulence grades providing an excellent starting biological material for molecular studies on MV strain differentiation and for the investigation of the molecular basis of virus attenuation in the field.

The aims of the study presented here were to molecularly characterise nine MV isolates [13] and to identify mutations that could be potential causes for virus attenuation. In carrying out this study we also aimed to identify genetic markers for the differentiation of MV isolates and to identify variable hotspots in the MV genomes therefore providing valuable tools for future epidemiological studies.

To accomplish these objectives preliminary RFLP analysis of purified full-length virus genomes and PCR-amplified terminal inverted repeats (TIR) regions were coupled with a comprehensive sequencing study of 16 genomic regions encompassing 20 genes (14 completely sequenced and 6 partially sequenced) and 7 intergenic regions. The sequenced regions covered approximately 10% of the virus genomes. The results of these analyses, together with the results from the published full sequence of the attenuated 6918 strain, are discussed with regard to MV strain differentiation, and potential causes for virus attenuation.

## Results

In order to identify regions of variability in the MV genome to be used for MV strain differentiation, phylogenetic studies and to investigate possible reasons for virus attenuation we endeavoured to molecularly characterise nine representative strains of MV from the 20 originally isolated in Spain between the years 1992 and 1995 [13] from wide ranging geographic locations (see Additional file 1). The virus strains had previously been characterised for their transmissibility and virulence in rabbits, with each strain given a virulence grade A-E [13], these grades being exactly equivalent to the grades I-V previously designated by Fenner and Marshall (1957). The selected strains were representative of different levels of virulence: 87, 466, 2012 and 86 (grade A); 2788, 7514 and 1312 (grade B); 7411 (grade C) and strain 4604 (grade E), which corresponded to a naturally attenuated MV isolate (See Additional file 2). It should be mentioned that although strain 6918 (grade E) was not directly analyzed in the present study the published sequence data [8] from the relevant regions of this strain were analyzed together with those from the 9 isolates studied. This was done because strain 6918 was originally isolated and characterized, in terms of virulence and transmissibility, under the same conditions [13] as the strains studied in this paper.

We selected 16 genome regions for sequence analysis (Table 1) based on the following criteria; 1) previous publications of MV strain characterisation [3,4,6-8], 2) publications identifying MV virulence factors [14-23], 3) RFLP analysis of genomic viral DNA and TIRs obtained using long-range PCR [24] (data not shown) and 4) proposed ORF functions [12].

**Table 1:** Primers used for the amplification of genes analysed in this study.

Gene	**Gene description**^**a**^	**Forward primer (5'-3') and genomic position**^**b**^	**Reverse primer (5'-3') and genomic position**^**b**^	Amplicon size (bp)	Reason for inclusion
**M002L/R**	TNF receptor homolog	GGTCCGTGATTAATATTCG 2864 - 2882/158892-158910^(c)^	GAATTCCACGCTGATGTAG 2121-2139/159635-159653^(c)^	761	Mutations detected in field strains [6]
**M004L/R**	ER localized apoptosis regulator	GGAATCTAGATAAGGAACATTG 4271-4292/157482-157503^(c)^	CGTCTTCCCGTAGAAGTC 5001-5018/156756-156773^(c)^	747	RFLP (data not shown)
**M004.1L/R**	Unknown				
**M005L/R**	Ankyrin-like				
**M007L/R**	IFNγreceptor homolog	CGAGGGTGATGTCGCTCATG 7891-7910/153864-153883^(c)^	GCGTACGCCACCTACCTCG 8835-8853/152921-152939^(c)^	962	Virulence factor [20]
**M009L**	Kelch ring canal protein homolog	CGCAGGTCCACGTATAAACC 11482-11501	ACGTGGGAAGCCGATGTC AG 13413-13432	1950	Mutation detected in strain 6918 [8]
**M010L**	EGF-like growth Factor	CGTTATGTGTACCGTATATG 13291-13310	GCGTGGGCACGTTGAACA CG 14180-14199	908	Virulence factors [15,18,20,22]
**M011L**	Integral membrane protein apoptosis regulator				
**M017L**	Unknown	CGTAGTAGTCAGAATCGTCG 17932-17951	CTATCCGGAAATCATAACC 18337-18355	423	Mutations detected in field strains [6]
**M034L**	DNA polymerase	GAACGAGGTTCGCATTGTAC 35048-35067	CTCTCAGGTGTTGAGTACG G 35691-35710	662	Potential diagnostic PCR
**M036L**	Leucine zipper motif	CAATCTAGCGTCAGATCCC 37128-37146	GTTTTCTGCGGATGGATCT C 39333-39352	2224	Mutation detected in strain 6918 [8]
**M069L**	Tyrosine/serine phoshatase	GAACTTAGAGTTGCTCATGCG 65999-66019	GAAGAAACAGACCGTGGA CAC 66714-66734	735	Mutation detected in strain 6918 [8]
**M071L**	Immunodominant envelope protein	CCATTGACTAACTCTGTTCC 67391-67410	CTAAATGGCGTCTCCTAGC 68165-68183	792	Gene function [12,17]
**M121R/M122R**	EEV glycoproteins	CAGACATCATGTCGTTACAC 115841-115860	CTTATAGAATCTTTTCATA C 116882-116901	1060	Gene function [12]
**M130R**	Virulence factor	GAGGATCATCCGAAGGAG 122677-122694	GGACTGTATATATCGCCTC 123373-123391	714	Virulence factor [21]
**M135R**	IL-1/IL-6 receptor-like	CCTACGTGTTTACTAGATTACG 131566-131587	GATTATCCTTCGTACGTCG 132290-132308	742	Mutation detected in strain 6918 [8]; Virulence factor [19]
**M141R**	Immunoglobulin domain	GAGAGACGATGCGTGTGTTAAG 137061-137082	GATGCATCGATTAACACGT C 137761-137780	719	Virulence factor [12]
**M144R**	Complement control protein homolog	CGTATCGGTTACGAAGAGTAG 139330-139350	CTAGATCGCCTCCTCTCCA GC 140369-140389	1059	Gene function [12]
**M148R**	Ankyrin-like	GCAAGCCGATGAGTTACTC 141533-141551	CGAATCCAGATTGTAGTAG 143727-143745	2212	Mutation detected in strain 6918 [8] Virulence factor [23]

^a ^[12]

^b^Nucleotide positions refer to myxoma virus Lausanne strain (Genbank accession no. AF170726)

^c ^Sequence occurs in the terminal inverted repeat regions

The sequences of the investigated genomic regions included 7 intergenic sequences and 20 complete genes or gene fragments, representing approximately 10% of the viral genome.

The sequence data from the 9 MV field strains indicated that two of them (466 and 2012) although isolated in very distant places in Spain (Additional file 1) could not be differentiated from one another and should be considered, with the available sequence data, as a single virus haplotype. All the remaining strains could be distinguised from each other using the obtained sequence data. The MV field strain sequences corresponding to the 16 amplified regions were aligned and compared to the published 6918 sequences (also isolated in Spain) and to those from reference strain Lausanne (Lu). Five of the regions analysed (corresponding to genes or gene fragments M034, M069, M071, M130 and M135) were identical (data not shown) in all the analyzed MV genomes.

In order to facilitate the description and analysis of the observed mutations in different MV isolates we have grouped the changes into three categories: those occurring within intergenic regions (Table 2), in the TIR (Table 3) or in central regions of the MV genome (Tables 4 and 5). The mutations are presented using the format; XpositionY, where X indicates the original Lu residue (GenBank accession number AF170726), position refers to the nucleotide number of the Lu sequence and Y indicates the mutation detected. For clarity the observed changes are described in separate sections.

**Table 2:** Mutations occurring within intergenic regions.

Gene		M002	M008.1	M017	M144
**Strain**^**a**^					
**A**	**87**	▼28 nt 2773	G11577A	▼8 nt 18224	
	
	**466**	Δ 16 nt (2773-2788)			
	
	**2012**	Δ 16 nt (2773-2788)			
	
	**86**				

**B**	**2788**	Δ 16 nt (2773-2788)			
	
	**7514**			G18210A	
	
	**1312**				

**C**	**7411**	Δ 28 nt (2773-2800)		Δ 16nt (18224-18239)	

**E**	**4604**			T18215C	A139394T
	
	**6918**				

The analyzed intergenic regions where those preceeding the indicated genes.

▼ insertion; Δ deletion. Nucleotide positions refer to the genomic positions in reference strain Lausanne (Genbank accession number AF170726). ^a^Strain virulence grade and identification number are indicated. Virulence grades being equivalent to the grades I-V previously designated by Fenner and Marshall [10].

**Table 3:** Mutations detected in TIR genes^a^.

Gene		M002		M004(f)		M004.1		M005(f)		M007	
										
Strain^b^		nt	aa	nt	aa	nt	aa	nt	aa	nt	aa
**A**	**87**					A4750G	F46L	C4937T	A483T	T8394C	Q128R
						G4754A					
	
	**466**			G4457C	N60K			C4937T	A483T		
	
	**2012**			G4457C	N60K			C4937T	A483T		
	
	**86**					A4750G	F46L	C4937T	A483T		
						G4754A					

**B**	**2788**	▼C 2600	FS			G4715A				T8394C	Q128R
	
	**7514**										
	
	**1312**	C2219T	D143N			G4775A				T8394C	Q128R
										G8456T	

**C**	**7411**	G2393A	R85W	C4412T							

**E**	**4604**										
	
	**6918**	C2594T	G18S							G8064A	S238F
		G2497A	S50F								

▼, insertion; nt, nucleotide residue; aa, amino acid residue; FS, predicted M002L ORF frame shift (^16^GGGAPYGADRGKCRGNDYEKD... to ^16^GGRCPVWRGSRKM*); * indicates STOP codon.(f) gene fragment.

^a^For clarity only the coordinates of changes occurring in the L-TIR genes are indicated. nt positions refer to the MV Lu genome (GenBank accession number AF170726).

^b ^Strain virulence grade and identification number are indicated. Virulence grades being equivalent to the grades I-V previously designated by Fenner and Marshall [10].

**Table 4:** Mutations detected in centrally located genes.

Gene		M009		M010		M011		M017		M036	
		nt	aa	nt	aa	nt	aa	nt	aa	nt	aa
**Strain**^**a**^											

**A**	**87**									▼T39162	FS5
										T38822C	K144E
										▼G38274	FS7
	
	**466**									▼TTT39162	▼K31
										T38822C	K144E
	
	**2012**									▼TTT39162	▼K31
										T38822C	K144E
	
	**86**									▼T 39162	FS5
										T38822C	K144E
										▼G38274	FS7

**B**	**2788**			C13481T	A55T	T13898C		▼TT18008	FS3	T38822C	K144E
				C13423T	S74N						
	
	**7514**	G12955A	T59M							▼TTT39162	▼K31
										T38822C	K144E
	
	**1312**	▼AT12172	FS1							▼TT39162	FS6
										T38822C	K144E
										Δ G38274	FS8
										A37515G	

**C**	**7411**	G13021A	S37L					Δ T18008	FS4	G39097A	A52V
										C38616T	

**E**	**4604**	C12818G	A105V			T13898C		▼TT18008	FS3	▼T39162	FS5
										T38822C	K144E
										G37215A	
	
	**6918**	Δ 10nt (11939-11948)	FS2							▼C37687	FS9
										C37733T	A507T
										T38701C	D184G
		C12293A	G280C							G38996A	R86W

▼, insertion; nt, nucleotide residue; aa, amino acid residue; FS, frame shift; * indicates STOP codon.

FS1, ORF changes from ^323^YVV... to ^323^YTS*; FS2, frame shift after aa 395 [8]; FS3, ^29^KRNRSVNN... to ^29^KKEIEV*; FS4, ^29^KRNRSVNN... to ^29^KEIEV*; FS5, ^29^KKHVSAILEFGF... to ^29^KKTCKRHFRIRVS*; FS6, ^29^KKHVSAILEFGF... to ^29^KKNM*; FS7, ^325^PPKLGESVSRKQSC... to ^325^PPQTGGIGEPKAIV*; FS8, ^325^PPKLGESVSRKQSC... to ^325^PPNWGNR*; FS9, frame shift after aa 446 [8]. ^a^Strain virulence grade and identification number are indicated. Virulence grades being equivalent to the grades I-V previously designated by Fenner and Marshall [10].

**Table 5:** Mutations detected in centrally located genes continued.

Gene		M121		M141		M144		M148	
		nt	aa	nt	aa	nt	aa	nt	aa
	**Strain**^**a**^								

**A**	**87**			T137666C				C142605T	A327V
									
									
	
	**466**							G141760T	E45D
									
	
	**2012**							G141760T	E45D
									
	
	**86**			T137666C				C142605T	A327V
									

**B**	**2788**	C115910A	S21Y					C142437T	A271V
								A142396G	
									
	
	**7514**			G137487A	R140Q	A139454G	Q15R	G141747A	R41Q
								C142651T	
									
	
	**1312**					C140154T			
									

**C**	**7411**					C140036T	A209V	A142396G	L142F
						C140143T	R245C	C142049T	
									

**E**	**4604**	C115910A	S21Y			C140143T	R245C	C142437T	A271V
									
	
	**6918**							G141940A	
								ΔC142964	FS9
									

▼, insertion; nt, nucleotide residue; aa, amino acid residue; FS, frame shift; * indicates STOP codon. FS9, frame shift after aa 446 [8]. ^a^Strain virulence grade and identification number are indicated.

Virulence grades being equivalent to the grades I-V previously designated by Fenner and Marshall [10].

### Sequence analysis of intergenic regions

The 7 intergenic regions analyzed corresponded to those preceding the ORFs M002, M004.1, M005, M008.1, M017, M122 and M144. Sequence data from all the MV strains investigated showed that the regions prior to ORFs M004.1, M005 and M122 did not have any mutations and only those preceding ORFs M002, M008.1, M017 and M144 had changes (Table 2) in some of the MV isolates.

**Figure 1:**
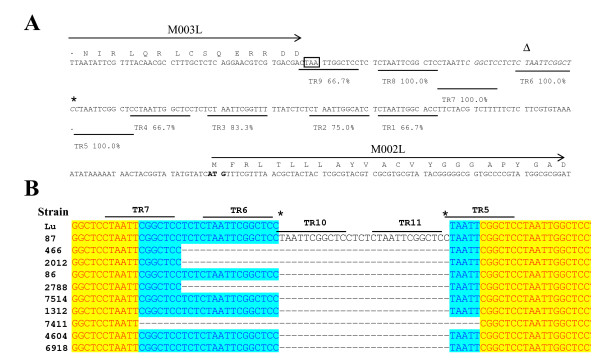
**A) Sequence of the intergenic region between genes M003L and M002L**. The sequence corresponds to MV Lausanne and is shown in the reverse complementary orientation. Amino acid sequences of M003 and M002 are indicated, the M003L stop (boxed) and M002L start (bold typeface) codons are also indicated. The nine tandem repeats (TR1-9) are marked and the percent homology to the canonical sequence (CTAATTCGGCTC) is shown. The sites of insertion (*) and deletions (Δ and italic typeface) are indicated. **B) Sequence alignment from the M002/M003 intergenic region**. Insertion and deletion mutations are shown and the relative TRs are indicated. The asterisks mark the site of the insertion of two copies of the TR (designated TR10 and TR11) in strain 87. Dashed lines represent sequences absent from particular strains. Highlighting is used to indicate degrees of homology between strains. The sequence alignment starts at nucleotide 2756 in Lu.

The intergenic region (178 nt) between ORFs M002 and M003 of MV Lu reference strain contains 9 copies of an imperfect tandem repeat (TR) of 12 nt (sequence CTAATTCGGCTC in the reverse complement of the genomic sequence) [6] and Figure 1A). Some of the analyzed MV field isolates (86, 7514, 1312, 4604 and 6918) also contained 9 copies of this repeat (Table 2 and Figure 1B). The remaining MV strains showed changes in the sequence of this intergenic region. Strains 466, 2012 and 2788 contained 8 copies of the TR sequence due to a 16 nt deletion in the TR6 region (Figure 1B). Strain 7411 had a 28 nt deletion which removed TR6 and half of TRs 5 and 7 and therefore contained 7 copies of the repeat whereas MV isolate 87 contained 11 copies of the repeated sequence (Table 2 and Figure 1B) due to an insertion of 28 nts at position 2773 occuring within the TR5 sequence (Table 2 and Figure 1B).

**Figure 2:**
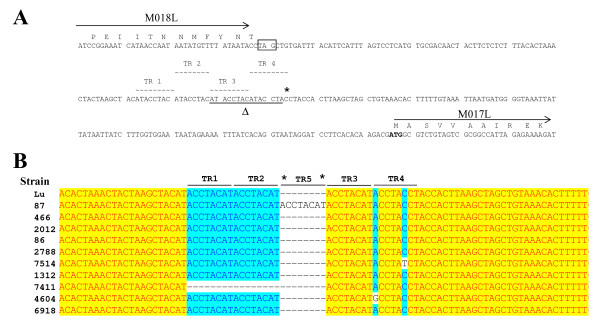
**A) Sequence of intergenic region between genes M018L and M017L**. The sequence corresponds to MV Lausanne and is shown in the reverse complementary orientation. Amino acid sequences of M018 and M017 are indicated, the M018L stop (boxed) and M017L start (bold typeface) codons are also indicated. The 4 identical tandem repeats (TR1-4) are shown (broken wavy lines). The sites of insertion (*) and deletions (Δ and underlined typeface) are indicated. **B) Sequence alignment from the M018/M017 intergenic region**. Insertion and deletion mutations are shown and the relative TRs are indicated. The asterisks mark the site of the insertion of the extra TR (designated TR5) in strain 87. Dashed lines represent sequences absent from particular strains. Highlighting is used to indicate degrees of homology between strains. The sequence alignment starts at nucleotide 18261 in Lu and the sequence is the reverse complement of the Lu published sequence.

The intergenic region between genes M017 and M018 contained 4 tandem repeats (sequence ACCTACAT in the reverse complement) in MV Lu (Figure 2A) and most of the strains analysed here (Figure 2B). Nevertheless, it should be mentioned that two of these field isolates (7514 and 4604) had a point mutation (Figure 2B) in TR4. The remaining two MV field strains had either insertions or deletions in this intergenic region. Isolate 87 contained an additional copy of this repeated sequence due to an 8 nt insertion following TR2 (Figure 2B) and strain 7411 had only 2 copies of this repeat (Table 2, Figure 2B) due to the deletion of TR 1 and TR 2.

The intergenic regions upstream of ORFs M008.1 and M144 were rather conserved in most MV strains and contained only single nucleotide point mutations in strains 87 and 4604 (Table 2).

### Sequence analysis of genes from within the TIRs

Considering the previous description of mutations in field strains as well as the virulence factor coding genes within the MV genome TIRs we decided to include three fragments from this region of the genome. For this purpose we designed three pairs of specific oligonucleotide primers (Table 1) which allowed the analysis of the full or partial sequences of 5 repeated genes (M002L/R, M004L/R, M004.1L/R, M005L/R, M007L/R) from within the TIRs (Table 3). For clarity only the coordinates of changes occurring in the L-TIR genes are indicated in the description (the numbering corresponds to the MV Lu genome GenBank accession number AF170726).

The M002 ORF sequence was conserved in six field isolates with respect to that of reference Lu strain. Strains 1312 and 7411 had nonsynonymous mutations at positions 2219 and 2393, affecting amino acid residues 143 and 85 of the corresponding protein products, respectively (Table 3). It should be mentioned that two different nonsynonymous mutations were also observed within this ORF (nt. 2497 and 2594) in the previously sequenced 6918 strain giving rise to non conservative amino acid substitutions at positions 18 and 50 (Table 3). In contrast to the reasonable conservation of this ORF in most of the studied field strains the 2788 isolate contained a single nucleotide insertion at position 2600 which led to a change in the amino acid sequence of the M002 ORF product from residue 18 onwards and to its truncation after residue 28 (Table 3).

Previous studies from our laboratory detected an *Mlu*I RFLP in several strains which mapped to the M004/M004.1/M005 genomic region (data not shown). The strategy used for sequencing of the entire M004.1L/R gene (Table 1) also led to partial amplification and sequencing of the M004L/R and M005L/R genes. In this region 7 different point mutations were observed in 7 of the 9 strains analysed (Table 3). Two isolates (4604 and 7514) did not have any changes with respect to reference strain Lu in the entire amplified M004/M004.1/M005 genomic region. This was also the case for the previously described strain 6918 although this last isolate had a C5009T mutation which mapped within ORF M005, outside the region sequenced in the present study. Strain 7411 contained only a silent mutation (C4412T) and isolates 466 and 2012 a nonsynonymous mutation (G4457C; N60K) in the sequenced M004 fragment. Four isolates (86, 87, 466 and 2012) contained a common nonsynonymous mutation (C4937T; A483T) in the sequenced M005 fragment, which led to the afore mentioned *Mlu*I RFLP (data not shown). In the fully sequenced ORF M004.1, isolates 87 and 86 shared two mutations one silent (G4754A) and one nonsynonymous (A4750G; F46L) whereas isolates 2788 and 1312 contained two other synonymous mutations (G4715A and G4775A respectively) not present in any of the other MV strains studied.

In the M007 gene most studied field strains (86, 466, 2012, 4604, 7411 and 7514) did not have mutations with respect to reference strain Lu. However, strains 87, 1312 and 2788 contained an identical nonsynonymous mutation (T8394C; Q128R) (Table 3), while strain 1312 contained a further silent mutation (G8456T) in this genomic region (Table 3). It should be mentioned that the published sequence of strain 6918 [8] also showed the presence of a nonsynonymous mutation (G8064A; S238F) within the M007 gene (Table 3).

### Sequence analysis of centrally located genes

In order to investigate putative changes in unique genes located in the central part of MV genome fifteen genes (M009L, M010L, M011L, M017L, M034L, M036L, M069L, M071L, M121R, M122R, M130R, M135R, M141R, M144R and M148R) were selected for PCR amplification using specific primers (Table 1) and nucleotide sequencing. Additional primers used for the sequencing of genes M009L, M036L and M148R are shown in Additional file 3. Five of these genes (M034L, M069L, M071L, M130R and M135R) were identical in the regions sequenced (data not shown) in all the analyzed MV genomes.

Four strains contained mutations in the M009 gene (Table 4). Strains 7514, 7411 and 4604 contained nonsynonymous point mutations at different positions, while strain 1312 contained a two-nucleotide insertion which led to disruption and truncation of the predicted ORF (Table 4). The analysis of the published M009L sequence from 6918 strain [8] showed the presence of a nonsynonymous point mutation (C12293A; G280C) and a 10 nt deletion which promoted a frame shift after amino acid residue 395 (Table 4).

Six genes of this central genome area (M010L, M011L, M121R, M122R, M141R and M144R) showed a low number of changes in some of the investigated strains (Tables 4 and 5). M121 had a single point mutation (C115910A; S21Y) in strains 2788 and 4604. M122 contained a single silent mutation (G116436A) in strain 2788. The M010L gene was conserved in all strains studied except for the 2788 isolate which contained two nonsynonymous mutations. M011L showed a single synonymous mutation which was shared by strains 2788 and 4604.

In the M141 region strains 87 and 86 contained a single silent mutation (T137666C) and strain 7514 a single nonsynonymous mutation (G137487A; R140Q). Strains 7514 and 7411 contained different nonsynonymous mutations within the coding region of M144 while strains 7411 and 4604 shared a nonsynonymous mutation (C140143T; R245C) in this gene. Strain 1312 was the only other strain to show a mutation in this region (C140154T).

Gene M148R showed sequence changes in most of the investigated strains, except for isolate 1312. M148 contained single nonsynonymous mutations in strains 86, 87, 466, 2012 and 4604 while three other isolates (2788, 7514, and 7411) showed two changes, a silent and a nonsynonymous mutation (Table 5). In the published 6918 sequence [8] M148R showed the presence of a silent mutation and a single nucleotide deletion (C142964) which promoted a frame shift.

Gene M017L which was conserved in most of the studied strains, contained insertions or deletions in three of the field isolates at genome position 18008 following a homopolymeric stretch of 8 thymidine residues. Strains 2788 and 4604 contained dithymidine insertions and strain 7411 contained a single thymidine deletion all promoting frame shifts and truncation of the resulting gene product (Table 4).

The M036L gene contained mutations in all nine strains studied when compared to the Lu published sequence. A single nonsynonymous mutation (T38822C) was present in 8 of the 9 strains, with strain 7411 the only strain not to present this mutation. Three strains (1312, 4604 and 7411) contained different synonymous mutations in this region and strain 7411 showed a nonsynonymous mutation (G39097A; A52V).

Two homopolymeric stretches within gene M036 were sites of multiple insertion or deletion mutations. Following a 7 nt homopolymeric stretch of thymidines, at base number 39162, three types of insertion were observed. Strains 87, 86 and 4604 contained single thymidine insertions, strains 466, 2012 and 7514 contained an insertion of three thymidines while strain 1312 contained an insertion of two thymidine residues. Following a guanosine homopolymeric stretch of 6 nts two strains (87 and 86) had single guanosine insertions (position 38274), while strain 1312 contained a single guanosine deletion (ΔG38274). The effects on the predicted ORF lengths of these insertions and deletions have on M036 are described in the footnote in Table 4.

## Discussion

Since the deliberate release of MV in Australia in 1950 and France in 1952 the emergence of more resistant hosts and the adaptation of virus strains has been well documented [25,26]. However, few studies have addressed the sequence changes that have occurred in the MV genome during this period [1,6,8,9] or more specifically the sequence changes involved in virus attenuation. Analysis of the molecular biology of MV in Australia have identified mutations in field strains [6] which have been used to study archived MV isolates and to track virus spread in the field [6,11]. Although attenuated strains were detected no correlation between the observed RFLPs and virulence was described. With the exception of the sequencing of the avirulent strain 6918 [8] and recent analyses of virulent MV circulating in Portugal and Spain [1,9] extensive studies on the molecular biology of European strains of MV are lacking, with sequence analysis concentrated on a single short fragment (491 nts) of the M022 envelope protein gene [3,4].

In order to bridge this gap in our understanding of the biology of MV we set out to molecularly characterise nine strains of MV isolated in Spain between the years 1992 and 1995. These strains were from wide ranging geographic locations from throughout Spain and had been previously graded for virulence by experimental infection of rabbits, and were chosen because they represent viruses with markedly different levels of attenuation [13].

To carry out epidemiological studies of MV it is essential to be able to differentiate between virus strains. Experimental infection of rabbits is not a suitable method for large scale continuous MV monitoring, therefore molecular characterisation of strains is preferable.

Using the mutations detected in this study the majority of the virus strains could be differentiated from one another. Despite a very high degree of sequence conservation, variable hotspots (regions containing M004L/R, M036L and M148R) in the genome were detected that could be used to provide phylogenetic data. We have not included a phylogenetic analysis using these fragments as the number of samples analysed was small (n = 9). However, the genetic relatedness of several strains could be inferred from shared mutations. In particular strains 466 and 2012, both virulence grade A [13] could not be differentiated from each other and shared 7 mutations with respect to the Lu sequence. Several strains also shared multiple mutations e.g. strains 87 and 86, and strains 2788 and 4604. These results may indicate that particular strains are derived from others or that the sites and type of mutations observed occur frequently. It is likely that some of the mutations arise frequently and revert showing the dynamic nature of the virus in the field. Further work will be required to determine if the mutations described are stable at the population level. Interestingly, the strains most related to each other originate in regions separated by between 500 and 1000 km (see Additional file 1). This observation is likely the consequece of movement of wild rabbits for repopulation of areas [27] rather than examples of convergent evolution.

The grossest changes were observed in the intergenic regions analysed. Deletions and insertions of tandem repeat sequences were common and have been reported in the Australian field studies [6]. These mutations are likely to be too frequent to be useful in phylogenetic studies.

With the exception of M036L, the highest numbers of mutations were observed in the terminal regions. Strains 87, 466, 2012 and 86 (all virulence grade A) contained numerous mutations in the TIRs and immediately flanking regions. Only strains 87 and 86 shared a further synonymous mutation in the central genome located in the M141R gene. However, the viruses in which we observed the highest numbers of mutations in the central regions were viruses that had reduced virulence grades when compared to Lu [13]. Strains 2788, 7514, 1312 (all grade B), 7411 (grade C) and 4604 (grade E) all contained mutations to genes in the central more conserved region of the genome. The presence of these mutations is surely indicative of the presence of more mutations yet to be detected, the cumulative effect of which may explain the attenuation of these strains.

In strain 6918 eighty mutations with respect to Lu were observed [8] however, it is not yet known which are responsible for the virus attenuation. Five 6918 genes carried mutations that disrupted ORFs (M009L, M036L, M069R, M135R and M148R). When the mutations found in the nine strains studied here and the strain 6918 are compared none were common. However, our data may provide insights into the reasons for virus attenuation of some of the nine strains analysed. Four genes contained mutations that disrupted ORFs, M002L/R, M009L, M036L and M017L.

M002 (previously termed MT-2) is a tumor necrosis factor receptor homolog with immunomodulatory functions [28]; reviewed by [29]. Inactivation studies have shown that M002 is a virulence factor [28]. Strain 2788 contained an insertion that disrupted the M002 ORF, and was somewhat but not completely attenuated with respect to Lu (strain 2788 is virulence grade B). While the analysis of individual genes may give us clues to the virulence of particular strains it is clear that factors such as compensatory mutations complicate the study of attenuation of viruses with large genomes.

While the function of M036 is unknown, disruptions to the M036 ORF occur in virulent ([6] and this work) and attenuated strains ([8] and this work) so it is unlikely that this mutation has an effect on virulence. It is clear that although the full gene product is not necessary for virus replication, it is maintained in the virus genome and therefore may offer variable regions to target to obtain phylogenetic data.

The M009L gene forms part of the interesting family of Kelch-like proteins represented in MV Lu genome by five genes (M006L/R, M008L/R, M009L, M014L and M140R) 3 as single copies and 2 duplicated at the left and right TIR. The M009 ORF is disrupted in the attenuated strain 6918 [8] and strain 1312. Morales et al., (2008) argue that this mutation is unlikely to be a critical virulence factor. In the study presented here, M009L was observed to contain a dinucleotide insertion that disrupted the ORF in strain 1312 and in virulence studies strain 1312 showed a somewhat reduced virulence (grade B, [13]) but was lethal in all rabbits infected. This could confirm that this ORF does not act as a critical virulence factor, however, until the appropriate knockout viruses are constructed and tested it cannot be ruled out that this protein plays some role in virulence.

The function of M017L is unknown. Nevertheless, considering the truncation of this ORF in strains 2788 and 4604 which showed reduced or complete lack of virulence respectively it is tempting to speculate that the truncation of this ORF, when coupled with the other mutations observed, may have an effect on virulence. Further work will be required to obtain the full-genome sequences and elucidate the exact role of the M017 protein in the virus life cycle.

It is unlikely that we have identified all the mutations responsible for the attenuation of the strains 7411 and 4604, virulence grades C and E respectively. Preliminary RFLP analysis of full-length genomes and TIRs amplified by long range PCR showed that no large deletions in the genomes of these viruses had occurred (data not shown). It is therefore likely that attenuation is due to the accumulation of point mutations and only full genome sequencing coupled with mutational analysis will show why these strains are less virulent.

Interestingly the most attenuated strain used in the study was strain 4604 which was isolated from the same region as strain 87, the most virulent isolate studied. No common mutations were identified between these strains in this study indicating that there are likely to be genetically distinct strains circulating in any one region. Repeated sampling will be required to show which strains predominate at present.

One of the most striking questions about the biology of MV is why should attenuated MV strains arise? The attenuation of some of the Spanish isolates may reflect a necessity for a tendency toward attenuation in geographical locations that had low density rabbit populations at the time of virus isolation [30]. Strains 7514, 1312 and 7411 all come from regions with low density wild rabbit populations. As MV has no other natural reservoir other than the European rabbit longer survival times in infected rabbits will increase the likelihood of encountering a new host or vectors in smaller populations. This is one aspect of virus/host evolution that could be examined using phylogenetic studies of MV isolates in Spain.

## Conclusions

In summary, the data included in this study provide an insight into the mechanisms of attenuation of MV strains and indicates useful targets for use in phylogenetic and epidemiological studies. The genes M004L/R, M036L and M148R were shown to contain variable sequences and can be considered possible targets for genetic relatedness studies. Using the type of data obtained in this study it will be possible to identify the types of virus circulating in an epidemic or outbreak, determine if new virus types are emerging or if one virus type is predominating in a particular area and be able to track the spread of these virus types.

## Methods

### Cells and virus

The nine MV strains used in this study were previously described and characterised for virulence in rabbits [13] (See Additional file 2). Each strain is identified by a number (assigned by [13]), region of origin in Spain (see Additional file 1) and virulence grade as follows: 87/Lleida/A, 466/Valencia/A, 2012/Asturias/A, 86/Badajoz/A, 2788/Albacete/B, 7514/Pontevedra/B, 1312/LaRioja/B, 7411/Canarias/C and 4604/Lleida/E. For simplicity, each strain is referred to by its identification number only from this point onwards. It should be mentioned that although strain 6918/Girona/E was not directly analyzed in this study the corresponding genome sequences from this virus isolate were also included in the analyses performed, together with the reference strain Lausanne (Lu).

*Myxoma virus *Lu strain and the nine field isolates listed above were grown and titered in RK13 cells which were maintained in Dulbecco's-modified Eagle medium (DMEM (Gibco, Carlsbad, CA)) supplemented with 10% foetal calf serum (FCS - PAA laboratories, UK) and 40 mg/L gentamicin (Gibco, Carlsbad, CA). To avoid the accumulation of mutations associated with virus adaptation to tissue culture the MV isolates were not passaged more than 3 times prior to genomic RFLP studies and were not passaged more than once for genome sequencing studies.

### MV DNA purification

For preliminary RFLP studies using full-length viral genomes we used the hypotonic burst method to rupture cells followed by PEG precipitation of virions as described previously [31]. Partially purified virions were treated with DNaseI (15 units, 1 h, 37°C; Fermentas). DNA was extracted from precipitated virions using a commercial kit (Masterpure™ Complete DNA and RNA purification kit, Epicentre^® ^Biotechnologies), following the total DNA purification protocol.

To obtain viral DNA for PCR and sequencing studies, total DNA was extracted from infected RK13 cell monolayers using the QIAamp DNA mini kit (QIAGEN, Hilden, Germany) as per the manufacturer's instructions.

The long-range PCR used for the detection of RFLPs in the TIR regions was previously described [24]. The oligonucleotides used for the PCR amplification of the MV genomic regions for sequence analysis are shown in Table 1. Additional primers used for the sequencing of genes M009L, M036L and M148R are shown in Additional file 3. The genes that were completely or partially sequenced were distributed (Additional file 4) along the full MV genome including both at right and left TIR and within the central not repeated sequences.

Cycle conditions were as follows; 94°C 2 min, then 35 cycles of 94°C 30 seconds, 55°C 30 seconds and 68°C 30 sec-2 min, with a final extension of 5-10 min at 68°C. Reactions were carried out using TaKaRa LA Taq (Takara, Madison, WI) in 1 × reaction buffer, 2.5 mM MgCl_2_, 2.5 mM of each dNTP, 2.5 units of enzyme and 0.2 μM of each oligonucleotide.

### DNA analysis, gels and software

Agarose (SeaKem Gold Agarose [Lonza, Basel, Switzerland]) gels (0.75%) in 0.04 M Tris, 1.14% acetic acid and 0.002 M EDTA (TAE) were run at room temperature and stained with ethidium bromide or with 1 × SYBR green stain (Invitrogen, Carlsbad, CA).

PCR reaction products were analysed by agarose gel electrophoresis. Full size PCR products were cut from gels using alcohol cleaned scalpel blades and purified using the Wizard SV gel and PCR clean-up system (Promega, Madison, WI, USA). Purified PCR products or MV genomic DNA were quantified on agarose gels by comparison with quantified DNA markers (GenerulerPlus, Takara, Madison, WI) and either subjected to digestions using a variety of restriction enzymes (as per manufacturer's instructions) for RFLP studies or 50 ng of DNA were mixed with 10 μM of the appropriate oligonucleotide and sequenced.

Agarose gels were photographed using the Gel logic 200I system, and images were analysed with Kodak Molecular Imaging software (Version 4), Kodak (NY, U.S.A).

### Sequence analysis

An ABI PRISM^® ^3130×l Genetic Analyzer (Sequencing services, Universidad de Oviedo) was used to sequence all gel purified PCR products and Chromas LITE freeware (Version 2.01- http://www.technelysium.com.au) was used to analyse sequences and to convert data to text format for subsequent alignment analysis.

Vector NTi version 11 (Invitrogen, Carlsbad, CA) software was used for all alignment analysis. The full-length sequences for Lu and strain 6918 were obtained from the NCBI database with the accession numbers AF170726 and EU552530 respectively.

## Competing interests

The authors declare that they have no competing interests.

## Authors' contributions

All authors read and approved the final manuscript. KD, JMMA and FP conceived the idea for the work. KD, IN and AB performed the analysis. KD and FP wrote the manuscript.

## Supplementary Material

Additional file 1 **Map of Spain showing the locations from where virus samples were collected **[13]. Diagram of Spain with the locations of virus isolations and the identification number of each virus indicated. Click here for file

Additional file 2 **Table showing virus strains, location of isolation, virulence grade and mortality**. Table adapted from the findings of Barcena et al., 2000, showing the identification number of virus strains, the virulence grades and mortality percentages. Click here for file

Additional file 3 **Table showing additional primers used in the sequencing of PCR products**. Additional primers used in the sequencing of large PCR products are shown. Click here for file

Additional file 4 **Schematic representation of the myxoma virus genes selected for sequencing**. The horizontal bar represents the myxoma virus genome (161 kb), while the vertical bars and arrows indicate the genes selected for analysis (Bars and arrows are to scale for the size of each gene). The terminal inverted repeats (TIRs) are labelled. Click here for file
