# Supplementary figures and images for: Molecular characterisation of virulence graded field isolates of myxoma virus
Source: Virol J. 2010 Feb 26;7:49. doi: 10.1186/1743-422X-7-49 (PMC2845566; doi:10.1186/1743-422X-7-49)

## Slide 1
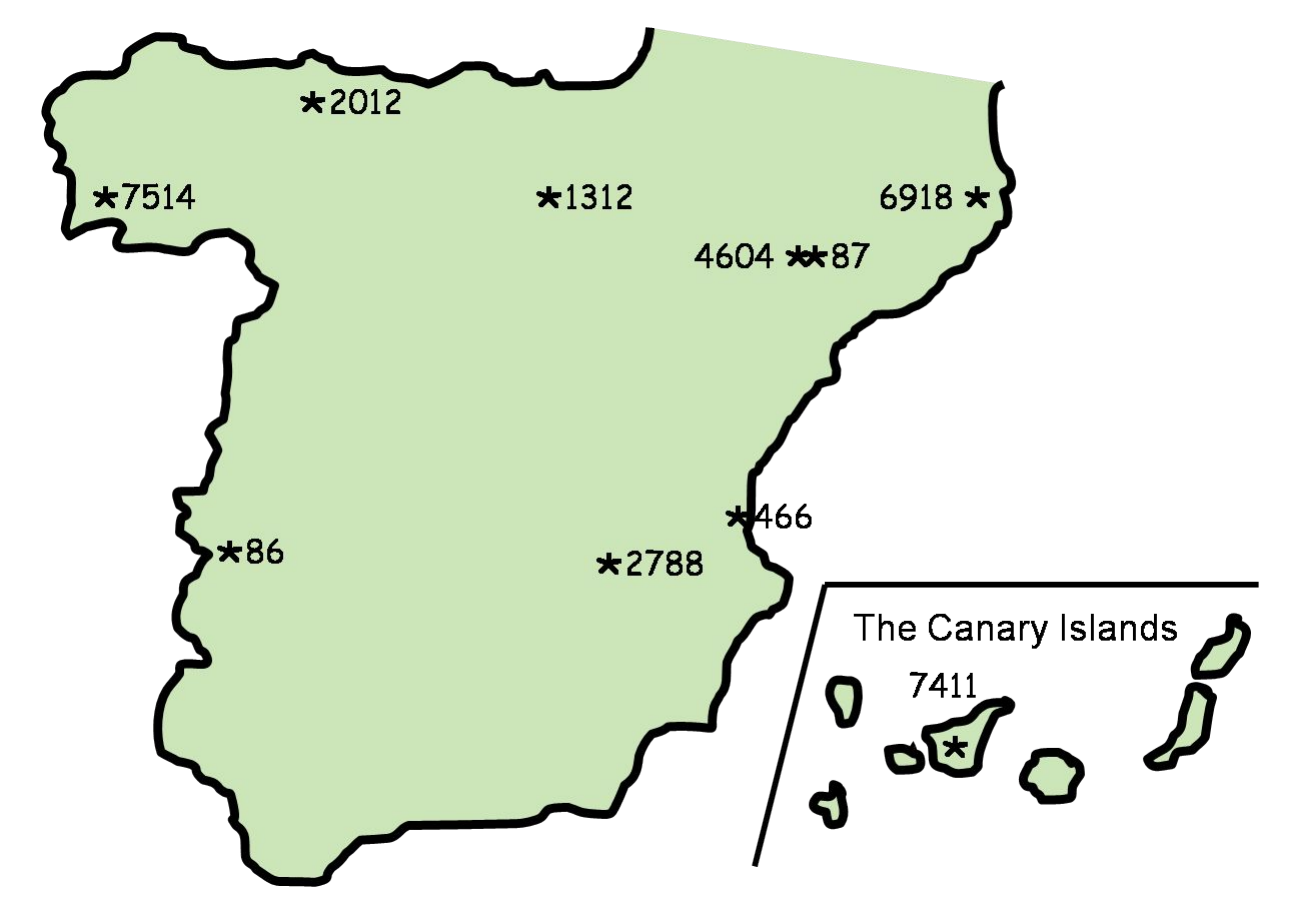

Supplement: Additional file 1 — Map of Spain showing the locations from where virus samples were collected [13]. Diagram of Spain with the locations of virus isolations and the identification number of each virus indicated. [file 1743-422X-7-49-S1.PPT]

## Slide 1
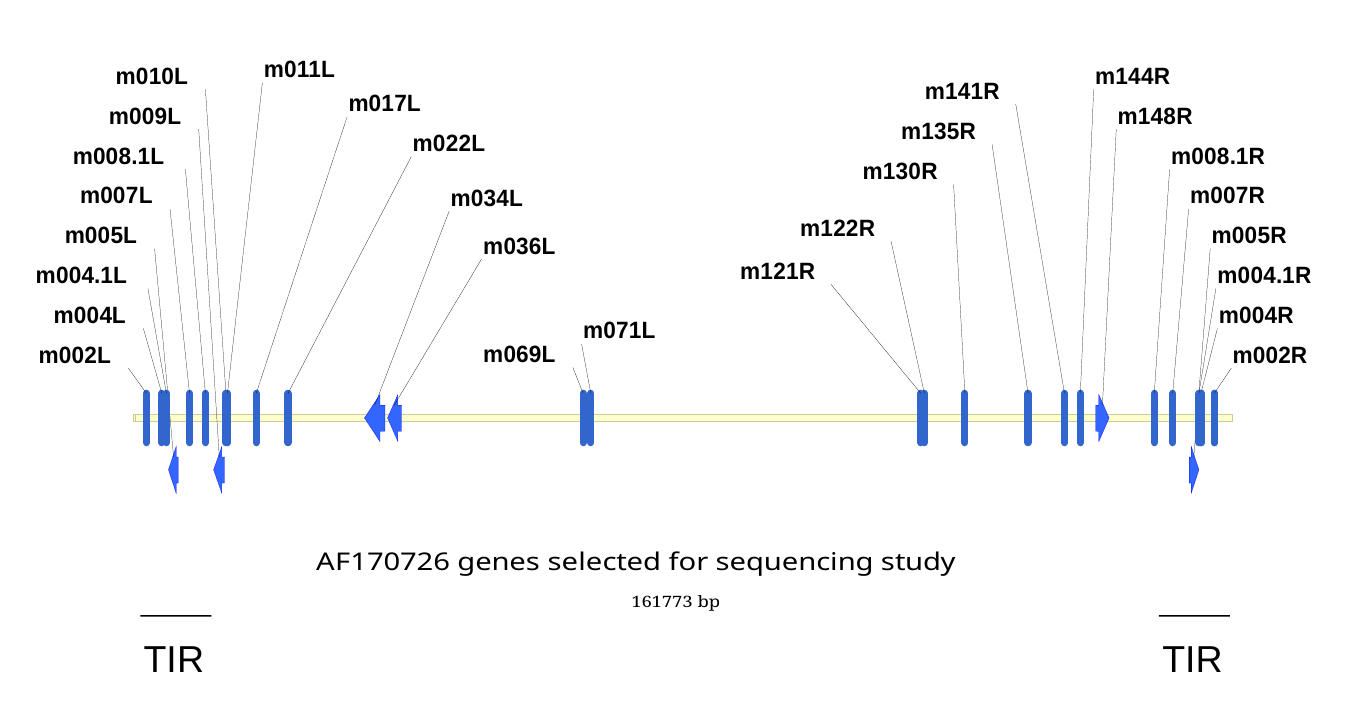

TIR
TIR

Supplement: Additional file 4 — Schematic representation of the myxoma virus genes selected for sequencing. The horizontal bar represents the myxoma virus genome (161 kb), while the vertical bars and arrows indicate the genes selected for analysis (Bars and arrows are to scale for the size of each gene). The terminal inverted repeats (TIRs) are labelled. [file 1743-422X-7-49-S4.PPT]
