# Supplementary material for: Molecular characterisation of virulence graded field isolates of myxoma virus
Source: Virol J. 2010 Feb 26;7:49. doi: 10.1186/1743-422X-7-49 (PMC2845566; doi:10.1186/1743-422X-7-49)
Supplement: Additional file 2 — Table showing virus strains, location of isolation, virulence grade and mortality. Table adapted from the findings of Barcena et al., 2000, showing the identification number of virus strains, the virulence grades and mortality percentages. [file 1743-422X-7-49-S2.DOC]

Additional file 2^a^ showing virus strains, location of isolation, virulence grade and mortality.

| **Strain** | **Location** | **Virulence grade^b^** | **Mortality** |
| --- | --- | --- | --- |
| 87 | Lleida | A | 100 % |
| 466 | Valencia | A | 100 % |
| 2012 | Asturias | A | 100 % |
| 86 | Badajoz | A | 100 % |
| 2788 | Albacete | B | 100 % |
| 7514 | Pontevedra | B | 100 % |
| 1312 | La Rioja | B | 100 % |
| 7411 | Canarias | C | 100 % |
| 4604 | Lleida | E | 0 % |
| 6918 | Girona | E | 0 % |

^a^ Adapted from [13].

^b^ Virulence grade is defined by the mean survival time of experimentally infected rabbits: Grade **A,** < 13 days (d); **B,** 13-16 d; **C,** 17-28 d; **D,** mortality < 50%; **E**, mortality 0%. Virulence grades being equivalent to the grades I-V previously designated by Fenner and Marshall [10].
