# Supplementary material for: Molecular characterisation of virulence graded field isolates of myxoma virus
Source: Virol J. 2010 Feb 26;7:49. doi: 10.1186/1743-422X-7-49 (PMC2845566; doi:10.1186/1743-422X-7-49)
Supplement: Additional file 3 — Table showing additional primers used in the sequencing of PCR products. Additional primers used in the sequencing of large PCR products are shown. [file 1743-422X-7-49-S3.DOC]

**Additional file 3. Additional primers used in the sequencing of PCR products.**

| **Gene^a^** | **Primer name** | **Primer sequence (5′−3′) and genomic position^b^** |
| --- | --- | --- |
| **M009L** | Seq9c  Seq9d | gttcgaaaatgtccagatcg  (12568-12587^c^)  ggttccatcgacatccatcg  (11932-11951) |
| **M036L** | Seq36c  Seq36d  Seq36e | Ctcttacggatacggagaaag  (38642-38662^c^)  Gtttaaactggatccgcgtatc  (38082-38103^c^)  cataccttcgataaacacg  (37815-37833) |
| **M148R** | Seq148c  Seq148d  Seq148e | ctagagtacgacgcatag  (143136-143153^c^)  gagtatatccctcataactgg  (142500-142520^c^)  cgtatcccaaactgaacatc  (142104-142123) |

^a^[12].

^b^Nucleotide positions refer to myxoma virus Lausanne strain (Genbank accession no. AF170726).

^c^Complementary with respect to the genome sequence AF170726.
